# Exploring Similarities and Differences of Non-European Migrants among Forensic Patients with Schizophrenia

**DOI:** 10.3390/ijerph17217922

**Published:** 2020-10-28

**Authors:** David A. Huber, Steffen Lau, Martina Sonnweber, Moritz P. Günther, Johannes Kirchebner

**Affiliations:** 1 Department of Forensic Psychiatry, Psychiatric Hospital, University of Zürich, 8006 Zurich, Switzerland; steffen.lau@puk.zh.ch (S.L.); martina.sonnweber@gmx.net (M.S.); johannes.kirchebner@puk.zh.ch (J.K.); 2 Department of Consultation-Liaison-Psychiatry and Psychosomatic Medicine, University Hospital Zurich, 8091 Zurich, Switzerland; moritzphilipp.guenther@usz.ch

**Keywords:** ethnicity, minorities, stigmatization, risk factors for criminal behavior, machine learning

## Abstract

Migrants diagnosed with schizophrenia are overrepresented in forensic-psychiatric clinics. A comprehensive characterization of this offender subgroup remains to be conducted. The present exploratory study aims at closing this research gap. In a sample of 370 inpatients with schizophrenia spectrum disorders who were detained in a Swiss forensic-psychiatric clinic, 653 different variables were analyzed to identify possible differences between native Europeans and non-European migrants. The exploratory data analysis was conducted by means of supervised machine learning. In order to minimize the multiple testing problem, the detected group differences were cross-validated by applying six different machine learning algorithms on the data set. Subsequently, the variables identified as most influential were used for machine learning algorithm building and evaluation. The combination of two childhood-related factors and three therapy-related factors allowed to differentiate native Europeans and non-European migrants with an accuracy of 74.5% and a predictive power of AUC = 0.75 (area under the curve). The AUC could not be enhanced by any of the investigated criminal history factors or psychiatric history factors. Overall, it was found that the migrant subgroup was quite similar to the rest of offender patients with schizophrenia, which may help to reduce the stigmatization of migrants in forensic-psychiatric clinics. Some of the predictor variables identified may serve as starting points for studies aimed at developing crime prevention approaches in the community setting and risk management strategies tailored to subgroups of offenders with schizophrenia.

## 1. Introduction

A growing number of studies demonstrate that immigration is not related to crime, yet the myth of the criminal immigrant is still influencing public debate and fuels many of the strategies pursued by political stakeholders [1,2]. The population of intercontinental migrants in Europe comprised approximately 38 million people in 2019 [3]. Before, during, or even after migration, some of these individuals have been exposed to traumatizing stressors, such as separation from social support, alienation, integration difficulties, or discrimination. Such stressors significantly increase the risk of mental illness [4,5,6,7]. With limited or no access to healthcare and healthcare systems involving numerous discriminatory practices, many migrants are prevented from receiving adequate treatment equal to that provided to patients without migrational backgrounds [8,9]. In addition, cultural and language barriers may prevent migrants from using mental health services or even basic medical aid services covered by mandatory health insurance [9,10,11]. This is particularly concerning, since research has demonstrated an increased risk for severe mental challenges in migrants. These include psychotic disorders or schizophrenia spectrum disorders (SSD), which are about twice as common among migrants compared to the non-migrant population and need comprehensive and professional treatment [12,13,14]. Psychotic disorders have disabling effects on the psychosocial functioning of the affected person and are also considered a risk factor for criminal and violent behavior, as indicated by major demographic and epidemiological studies [15,16]. In the face of the common prejudice that individuals with SSD are violence-prone, it should be noted that the absolute frequency of violent offences committed by individuals with a severe mental illness, including SSD, is rather low [16,17,18] with non-violent offences being more frequent than violent offences [19,20]. Studies conducted in Europe and in the United States demonstrated that migration does not constitute a risk factor for violent offending [21,22,23], but analyses on whether the positive correlation between SSD and violent behavior is stronger in ethnic minorities have yielded mixed results [24,25,26], which underlines the need for more research in this field. Some studies have found that forensic patients are more likely to be diagnosed with psychotic disorders, including SSD, if they have a migrant or ethnic minority background [24,27,28]. In this context, the role of ethnic variations in symptomatology of SSD remains to be clarified [29,30]. Cultural and linguistic differences between ethnic minority patients and mental health professionals have been reported to complicate diagnostic and therapeutic practice in both general and forensic psychiatry [10,31,32,33]. These challenges increase with the magnitude of the cultural differences between psychiatric professionals and patients. For example, intercultural differences between European nations can be assumed to be smaller than differences between an individual with a European cultural background and a non-European cultural background. On the other hand, the influence of cultural backgrounds during childhood on behavior in later life is fluid [34], which means that the effects of cultural background may diminish or accentuate after a change in cultural exposition or due to other life events, including migration. Effects due to migrational trauma versus cultural backgrounds may be difficult to discern. Overall, it seems difficult, if not impossible, to capture all of the inherent complexity in any study on the effects of migration or cultural differences, but a particular sensitivity to such complex aspects seems necessary. Most notably, the assessment of certain demographic factors as predictor variables for criminal behavior has led to controversial risk assessment methods such as racial profiling [35,36]. By exploring more sensitive predictor variables, controversial parameters such as “race” or “migration status” could possibly be replaced by less stigmatizing and less discriminatory ones. Given the overrepresentation of migrants and ethnic minorities in forensic hospitals compared to their relative proportion in the general population [37,38,39,40], this issue can be considered particularly relevant regarding forensic psychiatric settings.

In summary, a better understanding of criminal behavior among migrants with SSD could prove useful for risk assessment, preventive, and therapeutic purposes and also in combating stigma and discrimination against migrants. However, so far, a characterization incorporating socio-demographic, psychopathological, psychiatric, and criminological factors has not been conducted for this particular subgroup of offenders. Given this background, the aim of the present study is to apply machine learning (ML) to conduct a comprehensive comparison between native Europeans and non-European migrant offender patients with SSD detained in a Swiss forensic-psychiatric institution. While problematic, comparing native European offender patients with non-European migrant offender patients seemed the most practicable option to gain a glimpse on possible differences due to migration and cultural background. Since the foundation of this study is a complex database consisting of 370 patients and more than 500 variables, ML seemed to be most suited for this exploratory analysis. ML is a sub-form of artificial intelligence and relies on patterns and inference in a set of data in order to find an algorithm best predicting an outcome (such as European/non-European). In exploratory data analysis, it is therefore better suited than conventional statistical methods to uncover previously invisible non-linear dependencies between variables, often also resulting in better predictive power [41].

### Objectives

Employing ML algorithms, the objective of this exploratory study was to identify parameters that differentiate between offender patients with SSD of European versus non-European country of birth. Furthermore, we aimed at incorporating an extensive set of variables to identify the most influential of these variables and to quantify a predictive value for such differentiation.

## 2. Materials and Methods 

### 2.1. Source of Data and Measures

The files of 370 offender patients diagnosed with SSD as defined in chapters 295.0 to 295.9 of the ninth revision of the international classification of diseases (ICD-9) [42] and chapters F20.0 to F25.9 of the 10th revision of the international statistical classification of diseases (ICD-10) [43] who were admitted to the Center for Inpatient Forensic Therapies at the Zurich University Hospital for Psychiatry between 1982 and 2016 were analyzed retrospectively. The coding protocol covered the following domains: socio-demographic data, childhood/adolescence experiences, psychiatric history, past criminal history, social and sexual functioning, details on the offence leading to forensic hospitalization, prison data, particularities of the current hospitalization, and psychopathological symptoms by closely adopting the Positive and Negative Syndrome Scale (PANSS), whereby symptoms were divided into the usual 30 sub-categories and rated on a scale (completely absent, discretely present or substantially present). For full details on data collection and processing, see Kirchebner et al. [44] and Günther et al. [45]. 

### 2.2. Statistical Procedures—Machine Learning

**Figure 1:**
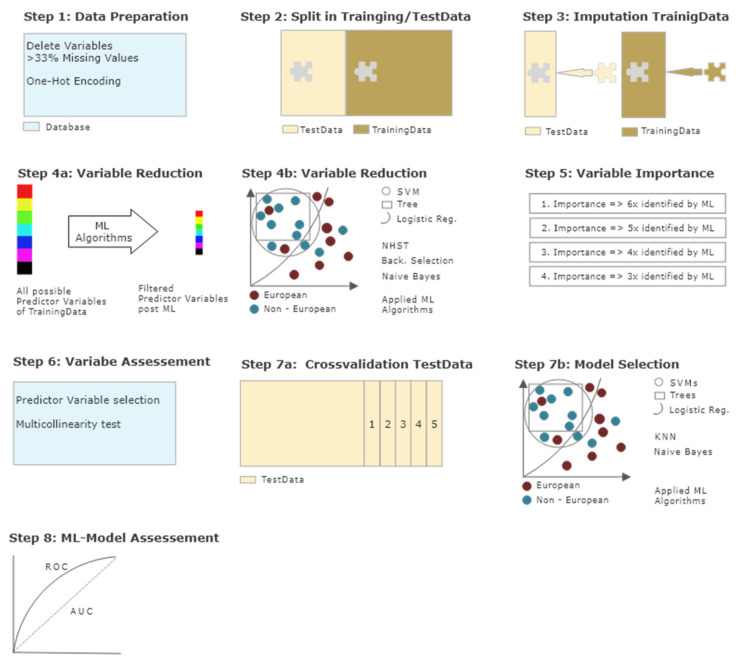
Data processing and statistical analysis. **Step 1**—Data preparation: Variables with more than 33% missing values eliminated; multiple categorical variables one-hot encoded; continuous variables not manipulated. **Step 2**—Data split: Split in training dataset with 70% of cases and test dataset with 30% of cases. **Step 3**—Imputation: Training and test dataset separately imputed via multivariate imputation by chained equations (MICE). **Step 4a**,**b**—Variable reduction: Identification of most influential variables in training dataset via different machine learning algorithms. **Step 5**—Variable importance: Variables ranked in order of importance, based on the number of times identified as most important by machine learning algorithms. **Step 6**—Variable assessment: Check for multicollinearity and selection of predictor variables identified at least 3x by algorithms. **Step 7a**,**b**—Model selection: Calculation and selection of best machine learning algorithm with identified predictor variables on test dataset embedded in 5-fold cross-validation. **Step 8**—Model assessment: Accuracy, area under the curve (AUC), sensitivity, specificity, positive predictive value (PPV), negative predictive value (NPV), extraction of best model.

Since this study was explorative in nature, supervised ML seemed to be the optimal method to identify the most important influencing factors of a multitude of variables and to determine the model with the best predictive power. An overview of all the statistical procedures can be seen in Figure 1 and a brief description is given below. For detailed information on ML in general and a more in-depth description of the different steps of our statistical approach in particular, see Kirchebner et al. [46].

All raw data was first processed for machine learning by transforming multiple categorical variables to binary code. Continuous variables were not modified. Variables with more than 33% missing values were deleted resulting in 653 variables. The outcome variable was defined as “patient with European country of birth (=European) / patient with non-European country of birth (=non-European)”. According to the European Migration Network (2018), there are complex often overlapping definitions of various subgroups of migrants [47]. For the purposes of the present study, we defined migrants as individuals who migrated to Switzerland for any reason from a non-European country (also see discussion section). Here, 266 patients (71.89%) were born in Europe, and 104 patients (28.11%) were not born in Europe. An overview of basic characteristics can be found in Table 1.

To combat the problem of overfitting, the original dataset of 370 patients was split into a training dataset with 70% of cases (259 patients) and a test dataset with 30% of cases (111 patients). Missing values were imputed separately for both datasets via 20 iterations of imputation using multivariate imputation by chained equations (MICE).

Then, variable reduction was performed on the training dataset, which aimed at identifying the most important predictors and served as a further measure against overfitting. Various statistical methods were utilized to ensure that the most important variables could actually be identified: null hypothesis significance tests (NHST; Fisher´s exact tests for categorical data; Mann-Whitney U-test for continuous data), backward selection, logistic regression (confidence interval of 99%), trees, support vector machines (SVM), and naïve Bayes. These variables were ranked in order of their importance, based on the number of times in which they were identified as most important by the above algorithms (i.e., variables identified as most important in all reduction algorithms; variables identified as most important in all but one algorithm, and so on). These most important variables were then checked for multicollinearity.

Finally, the test dataset was used to construct the best ML model with the variables identified above using different ML algorithms (trees, SVM, naïve Bayes, logistic regression and K-nearest neighbor). To counteract overfitting once again, this process was embedded in a five-fold cross-validation. The model with the highest accuracy was chosen as the final model, and goodness of fit was assessed using the receiver operating characteristic (ROC) curve method. Area under the curve (AUC) served as criterion to determine the level of discrimination. Additionally, specificity and sensitivity, positive predictive value (PPV), negative predictive value (NPV), and all confidence intervals (CI) were calculated.

### 2.3. Ethical Approval

This study was reviewed and approved by the Cantonal Ethics committee of Zurich, Switzerland (Ref. No. KEK-ZH-NR 2014-0480).

## 3. Results

All variables identified by the different algorithms as most important for distinguishing between European/non-European born patients are shown in the Appendix A.

Variables identified by at least three algorithms and thus ranking among the most relevant of the 653 potential predictors were chosen for the further model building. They belonged to the domains of socio-demographic data, childhood/adolescence experiences or particularities of current forensic hospitalization and are listed in Table 2. Variables of the domains psychiatric history, criminal past, social and sexual functioning, details of the crime that led to forensic hospitalization, prison data, or PANSS were not considered important by the algorithms employed. 

The two socio-demographic variables (migration, religious confession) were inevitably more present among non-Europeans, which consequently did not add any value to our research question and were therefore eliminated from further analysis. Multicollinearity tests did not show any irregularities. The absolute and relative distribution of the final variables used for model building can be seen in Table 3. 

**Figure 2:**
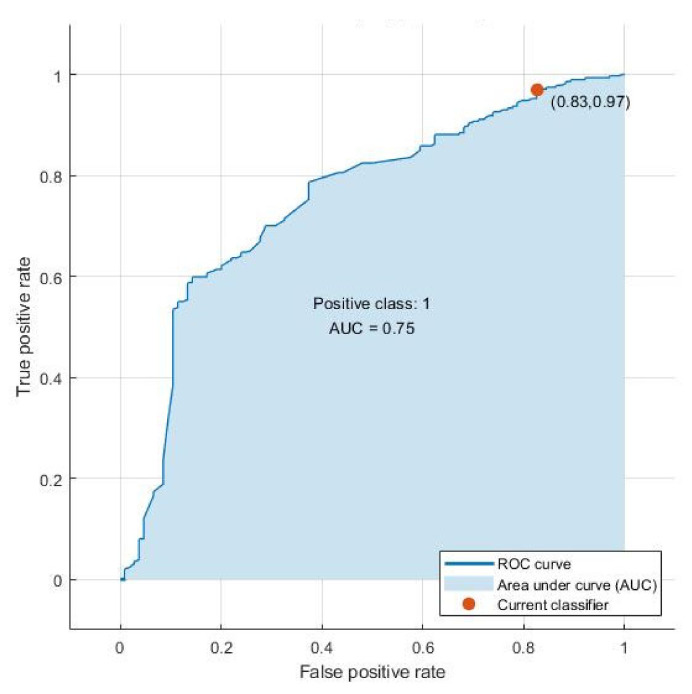
Receiver operating characteristic (ROC) curve of the final selected model (tree algorithm).

Using these predictor variables, a tree algorithm achieved the best results in distinguishing European/non-European patients with an accuracy of 74.5% and an AUC of 0.75 (see Figure 2). This model showed a sensitivity of 75%, reflecting its ability to correctly classify Europeans, and a lower specificity of 69%, reflecting its ability to correctly identify non-Europeans. The probability that the persons identified as European by the model were in fact European (positive predictive value, PPV) was 97%. The probability that the persons identified as non-European by the algorithm were actually European (negative predictive value, NPV) was 17% (see Table 4).

## 4. Discussion

Despite the increased prevalence of SSD in forensic patients with a migrational or ethnic minority background [24,27,28], research on how this offender subgroup differs from other offenders with SSD is scarce. In the present study on this subgroup, the combination of only five variables (mean dose equivalent of olanzapine at discharge, patient suffering from poverty in childhood/adolescence, social isolation in childhood/adolescence, only engaged in most basic tasks in ergotherapy, language problems during psychotherapy) allowed us to distinguish between native European and non-European offenders with SSD with a significant predictive power (AUC = 0.75). Interestingly, the majority of factors examined, which include all of the criminal and psychiatric history factors, the type of offence that led to the forensic hospitalization, and PANSS, were not identified as essential for model building. With regard to PANNS, which was evaluated at both admission and discharge from the forensic-psychiatric clinic, neither the individual items nor the positive, negative or total score showed marked differences between the two groups studied. Overall, these results suggest that the subgroup of non-European migrants may be quite similar to the rest of the offenders with SSD, except for a few differences to be discussed in more detail below. The childhood/adolescence variables “patient suffering from poverty” and “social isolation” were among the variables with the highest predictive power, which could be explained by the pronounced group differences in these two factors. More specifically, 59.1% of the non-European offender patients grew up in a family suffering from poverty compared to 31.3% of the native European individuals. In contrast, only 27.6% of migrant patients suffered from social isolation during childhood/ adolescence, compared to 57.3% of native Europeans. These group differences may be attributable to the origin of migrants from countries with poor economies, but with collectivist cultures and closer family structures, since the majority of migrant patients came from Africa and the Middle East. More importantly, however, previous research suggests that childhood poverty is associated with an increased risk for delinquency that extends into adulthood [48,49,50] and that social isolation in childhood and early adolescence constitutes a risk factor for juvenile violence [51,52]. Therefore, the pronounced group differences in the two childhood/ adolescence variables may be relevant in a criminological context and it would be desirable to understand the pathways to criminal behavior associated with these two factors, as this could prove useful in developing risk prevention strategies tailored to subgroups of offenders with SSD. Previous research suggests that people with SSD may develop criminal behavior not only on the basis of psychotic symptoms but also in parallel or even independently of psychosis [53,54,55]. Based on the findings of our analysis and previous research, we assume that differences in criminal behavior between European and non-European patients may be largely attributable to psychosis-independent factors. More specifically, if the two identified childhood/adolescence factors (or other undetected group differences) are related to criminal behavior that is mediated by psychosis, one would expect differences in psychiatric history or psychotic symptomatology between the two groups. However, our analysis neither found distinct group differences in the psychiatric history nor in the PANSS at admission or discharge. Additionally, previous research has shown that poverty and social isolation in childhood are risk factors for criminal offenses, which are not limited to individuals with psychosis [48,49,50,51,52]. We therefore assume that in the development of criminal behavior, psychosis is not a relevant moderator or mediator variable with regard to the two childhood factors we have identified to be good predictors of subgroup allocation. It is rather an indication that many migrants have suffered an accumulation of (early) traumatic experiences, which has been shown to be a major risk factor for violence and offending [44,56], and that neither origin or ethnicity nor psychosis determine criminal behavior.

Our analysis also revealed group differences in three therapy-associated factors related to the forensic-psychiatric environment, specifically, that non-Europeans had more “language problems during psychotherapy”, that they were more often “only engaged in the most basic tasks in ergotherapy”, and that the group of migrant patients received a higher “antipsychotic dose equivalent to olanzapine at discharge”. The group differences in those factors might be attributed to cultural and linguistic misunderstandings between migrants and mental health professionals, an issue which has been described in the clinical practice of both general and forensic psychiatrists [10,31,32,33]. Based on this assumption, a lack of cultural and/or linguistic competency may have led mental health professionals to misjudge the potential skills and resources of migrant patients as well as their health status. As a result, clinicians have assigned them simpler, less demanding tasks in ergotherapy and prescribed higher doses of antipsychotic drugs. It is also possible clinicians confused language barriers or respectful shyness with negative symptoms of SSD. Earlier studies even found that language competence is related to the duration of inpatient psychiatric treatment [57] and that the quality of language skills plays a crucial role in the subjectively experienced distress load [58]. However, since the PANSS was only assessed at admission and discharge, it cannot be completely ruled out that the migrant patients received higher doses of antipsychotic drugs because of exhibiting more symptoms during hospitalization.

Finally, it must be noted that ML cannot be used as a possible automated process for assigning psychiatric patients to groups (e.g., migrants/non-migrants, dangerous/non-dangerous) or even trying to predict their future behavior. Models with sensitivity and specificity below 100% will cause patients to be mistakenly assigned to the wrong groups or false predictions will be made. Given such sensitive areas involving human life, treatment, and stigmatization, an intensive discourse is needed on how data can be collected and used and what criteria must be met by ML models to be considered useful. In this study, ML should be seen as an advanced statistical method for retrospective differentiation of individuals rather than as a predictive modeling technique. Our results can be used as a guide to optimize existing risk assessment and treatment but needs to be examined in further studies, ideally in a prospective manner.

### 4.1. Strengths and Limitations

A major strength of this study is the examination of 370 individuals of a specific subgroup using an extensive range of variables in a rather unexplored field. ML algorithms enabled an exploratory data analysis of 653 potential predictor variables, including various socio-demographic, psychopathological, personal, and criminal history factors. To date, there appears to be no other study on offenders suffering from SSD that has compared characteristics of European and non-European patients based on such a variety of variables. The multiple testing problem was minimized by excluding predictor variables which could not be cross-validated by at least three out of six different machine-learning algorithms. Therefore, despite the large number of comparisons between the two groups, the significance of the detected predictor variables is emphasized by a multiple validation procedure.

Nevertheless, the present study also has several limitations. To obtain sufficient amounts of data, files from a period of more than 30 years were collected and reviewed. Given this long period of time, it cannot be excluded that intracultural changes within the study population and intercultural changes between the study population and the treating mental health professionals may have influenced the results of our study. Also, no comparisons within groups were made. In particular, the different ethnicities within the group of migrants were not compared, as the migrant sample comprised 104 persons in total, which was considered too small for a conclusive group analysis. In addition, differentiating factors between European and non-European offender patients due to cultural background were not discerned from those due to traumas experienced before, during or after migration. Furthermore, individuals born/not born in Europe (i.e., definition of the outcome variable) cannot be set equal to individuals with/without refugee status or a history of forced migration or immigration. On the one hand, there may be forced migration of patients born in Europe, and on the other hand, the patients not born in Europe compose a heterogeneous subgroup of individuals with diverse and distinct cultural and individual backgrounds and pathways leading to their migration. However, based on the data available to be analyzed, the definition of the outcome variable chosen here seemed the best approximation available to capture patients with a history of major relocation and a cultural background foreign to Switzerland.

Retrospective data analysis involves the risk of inconsistencies in data collection and despite quite extensive criminal records, not every variable could be collected from every patient. This problem was addressed by using imputation algorithms for variables with less than one-third missing values and by excluding variables with more than one-third missing values from the analysis. However, more consistent data would have been desirable to better prevent possible bias. Since the study was conducted in a single forensic psychiatric center in Switzerland, it is unclear how well the results are generalizable to other hospitals, other countries, and other healthcare systems. Replication studies in different settings would contribute to clarify this question. 

### 4.2. Implications—Reduction of Stigma and Discrimination

Forensic patients with migrational history belonging to ethnic minorities suffer from multiple stigmatization due to their minority status, mental illness, and criminal record [59]. The finding that forensic patients with SSD of European and non-European origin are similar with regard to their criminal past, types of offences that led to their forensic hospitalization, and psychopathological profile may help to combat stigmatization and discrimination of these individuals. 

### 4.3. Implications – Risk Prevention

With regard to risk prevention, our analysis highlights two known predictor variables (“patient suffering from poverty in childhood/adolescence” and “social isolation in childhood/adolescence”) [48,49,50,51,52] that may be largely or entirely independent of psychosis, thus supporting studies suggesting that offences in schizophrenia do not necessarily have to be associated with psychosis [53,54,55]. In light of this, the present study emphasizes that the prevention of criminal behavior among people with SSD should not focus solely on reducing psychotic symptoms, but rather requires multimodal prevention strategies that also target risk factors for crimes beyond psychosis. This finding is in line with previous research, which demonstrated that the deinstitutionalization of mentally ill individuals does not correlate with offences which lead to imprisonment [60,61,62]. Rather, it appears that the incarceration of mentally ill people is related to a lack of outpatient support, such as community-based services and opportunities for supported employment and sheltered housing [63,64]. They are confronted with various challenges beyond their symptoms that may affect their behavior and that cannot be adequately treated either with psychotherapy or with pharmacotherapy. Our analysis indicates that growing up under adverse circumstances such as poverty or social isolation should not be underestimated as a risk factor for criminal behavior, even for people with conspicuous psychopathological symptoms such as psychosis. This finding might be of particular relevance when considering that over 20% of children were at risk for poverty or social exclusion in the European Union in the year 2019 [65]. In order to reduce crime rates in individuals with SSD, but also in general, it might thus be worthwhile to implement prevention measures which tackle poverty and social isolation in the community setting from an early age on and before the potential onset of a mental illness.

### 4.4. Implications—Diagnostics, Therapy, and Risk Assessment

In the forensic-psychiatric context, erroneous assessments of a patient’s mental health can have far-reaching consequences with regard to diagnostics, therapy and risk evaluation [33,66]. Previous authors have emphasized the importance of acquiring cultural competence for health professionals, with some suggesting that mental health experts in forensic-psychiatric clinics could work with interpreters or cultural mediators to better avoid misunderstandings and errors [66,67,68]. Our analysis supports such approaches and suggests that better linguistic understanding and cultural sensitivity among psychosocial professionals could help to provide treatment that is better tailored to the needs and resources of migrants with SSD. This is particularly important with regard to the prescription of higher doses of antipsychotic drugs to migrant patients, which indicates that the separation of cultural idiosyncrasies and psychopathology may pose a particular challenge in this regard. Identifying and trying to avoid this kind of confusion can be crucial in a system where equality in therapy and equality before the law should be guaranteed.

## 5. Conclusions

The potential of machine learning for exploratory analysis of patient data is increasingly recognized in psychiatric research [44,69,70]. Its applicability in the forensic-psychiatric context is supported by the present study, which investigated similarities and differences between native Europeans and non-European migrants on a sample of 370 criminal offenders with SSD. Given the frequent stigmatization of migrants detained in forensic psychiatric institutions [59], it is important to stress that the group differences we identified were outweighed by a large number of similarities. Of special importance is the finding that forensic patients with SSD of non-European origin do not commit particularly serious types of offences or exhibit particularly severe psychopathological symptoms compared to forensic patients with SSD of European origin. Nevertheless, certain group differences are worth mentioning, as they could prove useful for the implementation of risk prevention approaches in the community setting as well as for the development of risk assessment and risk management strategies that are better tailored to certain subgroups of offenders [35,55]. In particular, our analysis revealed group differences in the variables related to childhood/adolescence. Assuming that the groups thus differ in their pathways to criminal behavior, it is likely that these are not generally related to psychosis, since no pronounced group differences were found in neither psychiatric history factors nor symptomatology. Apart from this, our analysis has revealed group differences associated with the forensic-psychiatric therapy setting, which may at least partly be attributable to cultural and linguistic misunderstandings between migrants and mental health professionals. However, since the exploratory research design does not allow for definite inferences, we encourage scholars in this field to conduct hypothesis testing studies based on the findings of our exploratory study. In this sense, we strongly recommend paying attention not only to differences, but also to similarities between migrants and natives, as the reduction of stigma should not be neglected in favor of the development of potentially stigmatizing risk prevention strategies. 

## Figures and Tables

**Table 1:** Description of study sample.

Variable	Total N = 370	European Nationality N = 266	Non-European Nationality N = 104
Male sex	339 (91.6)	243 (91.4)	96 (92.3)
Age at admission (mean, SD)	34.15 (10.226)	34.37 (10.695)	33.60 (8.942)
Single (at offence)	297 (80.3)	224 (84.2)	73 (70.2)
Diagnosis schizophrenia (ICD-9/10)	294 (79.5)	219 (82.3)	75 (72.1)
Birth country Switzerland	167 (45.1)		
Other European country	99 (26.8)		
Middle East	25 (6.8)		
Africa	54 (14.6)		
Other Country	25 (6.8)		

*Note*. SD = Standard deviation.

**Table 2:** Most important variables to differentiate between European/non-European identified by at least three algorithms.

Variable Description	Variable Code	Frequency of Identification
Mean dose equivalent of olanzapine at discharge	R9e	5
Religious confession of the patient: Islam	SD4b	5
Migration experienced	SD20	5
Patient suffering from poverty in childhood/ adolescence	CJ16	4
Social isolation in childhood/ adolescence	CJ1	4
Only engaged in most basic tasks in ergotherapy	R17b	3
Language problems during psychotherapy	R16g	3

**Table 3:** Absolute and relative distribution of predictor variables.

Variable	European n/N (%)	Non-European n/N (%)
Mean dose equivalent of olanzapine at discharge (with SD)	18.46 (12.93)	21.22 (16.52)
Patient suffering from poverty in childhood/ adolescence	68/217 (31.3)	39/66 (59.1)
Social isolation in childhood/ adolescence	126/220 (57.3)	16/58 (27.6)
Only engaged in most basic tasks in ergotherapy	110/263 (41.8)	70/102 (68)
Language problems during psychotherapy	3/265 (1.2)	10/97 (10.31)

*Note*. SD = standard deviation.

**Table 4:** Final tree model performance measures.

Performance measures	% 95% Confidence Interval
Accuracy	74.46 [69.92, 78.76]
AUC	0.7500 [0.5389, 0.8610]
Sensitivity	75.00 [70.01, 79.42]
Specificity	69.23 [48.10, 84.91]
PPV	96.99 [93.94, 98.59]
NPV	17.31 [10.85, 26.24]

*Note.* AUC = area under the curve (level of discrimination); PPV = positive predictive value; NPV = negative predictive value.

## Appendix A

**Table A1:** Predictor variables identified by at least one machine learning algorithm.

Variable Code	Variable Description	Algorithm
SD4a	religious confession of the patient: Catholic	NHST, naive Bayes
SD4b	religious confession of the patient: Islam	NHST, backward selection, trees, SVM, naive Bayes
SD5a	marital status	NHST
SD14	own children	NHST, tree
SD18a	legal guardian: birth parents	NHST, SVM
SD18b	legal guardian: single parent	tree
SD20	migration experienced	NHST, backward selection, trees, SVM, naive Bayes
CJ1	social isolation in childhood/ adolescence	NHST, backward selection, logistic regression, tree
CJ8	professional help by a psychiatrist/psychologist sought in patient´s childhood/ adolescence	NHST
CJ12	severe conflicts between the parents in patient´s childhood/ adolescence	NHST, logistic regression
CJ16	patient suffering from poverty in childhood/ adolescence	NHST, backward selection, tree, SVM
CJ29	school failure	NHST, logistic regression
PH4	symptom hallucinations in patient’s history	NHST
PH14c	opioid abuse/dependency in patient’s history	NHST
PH14d	cocaine abuse/addiction in patient’s history	NHST
PH14e	stimulants, amphetamines, ecstasy abuse/addiction in patient´s history	NHST
PH17a	persistent refusal of occupational therapy during inpatient treatment	NHST
PH18a	outpatient psychiatric treatment(s) before investigated offence	NHST
PH19c	number of inpatient treatment(s) before investigated offence	NHST
PH19d	time period between release out of last inpatient treatment und the investigated offence in weeks	SVM, naive Bayes
PH25e	homeless at time of the investigated offence	NHST
CH4k	criminal record: traffic offence	NHST, backward selection
CH10b	stay in forensic institution mandated by jurisdiction	NHST
S4	psychosocial variable: low self esteem	NHST
J1	time spent in prison	Tree
J3b	type of imprisonment	Logistic regression, SVM
D1	amount of index offences	SVM, naive Bayes
D2g	index offence: property crime without violence	NHST
D2k	index offence: traffic offence	NHST
D10a	in relationship with victim	NHST, backward selection,
D11c	location of offence: mutual home with victim	NHST, SVM, naive Bayes
D11j	location of offence: public space	NHST
D22b	patient´s subjective statements correspond with the facts described in the police records	NHST
D25a	patient touched victim’s genital/breast	NHST
D25b	patient performed vaginal intercourse at offence	NHST
R1c	current psychiatric F2X diagnosis - acute psychotic disorder	NHST
R4d	assigned from prison	NHST
R5	patient in legal measure	NHST
R8l	clozapine medication in current hospitalization	NHST
R9e	mean dose equivalent of olanzapine at discharge *	Backward selection, logistic regression, tree, SVM, naive Bayes
R15a	main content of psychotherapy/ conversations “more loosening within forensic setting”	NHST
R16g	language problems during psychotherapy	NHST, tree, naive Bayes
R17b	only engaged in most basic tasks in ergotherapy	NHST, backward selection, SVM
R19	insight into wrongfulness of offence	NHST
R22a	time spent in current forensic hospitalization (weeks)	NHST
R25b	Presumably close contact to family at release of current forensic hospitalization	NHST, backward selection
R26	insight into illness and its treatment	NHST
PANSS3	PANSS at admission: Scale Hallucinations	NHST
PANSS23	PANSS at admission: Scale Unusual thought content	NHST

*Note.* NHST = null hypothesis significance testing; SVM = support vector machines; PANNS = Positive and negative syndrome scale; * Note that for conversion of cumulative antipsychotic dosages into olanzapine equivalents, conversion factors provided by the classical weighted mean dose method [71] were used with a very small number of exceptions where older antipsychotics were prescribed. In these cases, the minimum effective dose method [72] or international experts’ consensus-based olanzapine equivalents [73] provided the necessary converting factors.
